# Molecular Regulatory Mechanism of Exocytosis in the Salivary Glands

**DOI:** 10.3390/ijms19103208

**Published:** 2018-10-17

**Authors:** Akiko Suzuki, Junichi Iwata

**Affiliations:** 1 Department of Diagnostic & Biomedical Sciences, The University of Texas Health Science Center at Houston School of Dentistry, Houston, TX 77054, USA; akikosuz925@gmail.com; 2 Center for Craniofacial Research, The University of Texas Health Science Center at Houston School of Dentistry, Houston, TX 77054, USA; 3 Program of Biochemistry and Cell Biology, The University of Texas Graduate School of Biomedical Sciences at Houston, Houston, TX 77030, USA

**Keywords:** exocytosis, salivary glands, Sjögren’s syndrome, protein secretion, membrane trafficking

## Abstract

Every day, salivary glands produce about 0.5 to 1.5 L of saliva, which contains salivary proteins that are essential for oral health. The contents of saliva, 0.3% proteins (1.5 to 4.5 g) in fluid, help prevent oral infections, provide lubrication, aid digestion, and maintain oral health. Acinar cells in the lobular salivary glands secrete prepackaged secretory granules that contain salivary components such as amylase, mucins, and immunoglobulins. Despite the important physiological functions of salivary proteins, we know very little about the regulatory mechanisms of their secretion via exocytosis, which is a process essential for the secretion of functional proteins, not only in salivary glands, but also in other secretory organs, including lacrimal and mammary glands, the pancreas, and prostate. In this review, we discuss recent findings that elucidate exocytosis by exocrine glands, especially focusing on the salivary glands, in physiological and pathological conditions.

## 1. Introduction

The major (submandibular, sublingual, and parotid) and minor salivary glands produce 0.5–1.5 L of saliva daily, which is essential for oral health. Saliva comprises 99.5% of water, 0.3% of proteins, and 0.2% of both inorganic and organic substances [1]. The solid components differ from person to person, and from time to time in the same person. For example, salivary glands secrete 1.5 to 4.5 g proteins daily at a concentration of 0.47 ± 0.19 to 2.67 ± 0.54 mg/mL [2]. The protein concentration in saliva is lower than that in tears, which is around 8 mg/mL under basal conditions [3,4]. Acinar cells are a major cell type within the salivary glands, responsible for the production and secretion of prepackaged secretory granules that contain key functional salivary components, such as amylase, mucins, and immunoglobulins [5]. These salivary components are functionally important for the digestion and taste of foods, lubrication, buffering, and prevention of dental caries, periodontitis, candidiasis and halitosis (bad breath).

This secretion process in exocrine glands, called exocytosis, involves secretory vesicle trafficking, docking, priming, and membrane fusion [6]. A failure during any of the steps in exocytosis in the salivary glands results in altered secretion of salivary proteins (Table 1). Interestingly, several studies show that secretion of salivary proteins is reduced, and the contents of saliva are also changed, in patients with dry mouth syndromes (e.g., Sjögren’s syndrome) and in a mouse model for Sjögren’s syndrome (e.g., non-obese diabetic (NOD) mice) [1,7,8,9,10]. This evidence suggests that exocytosis may be altered in various conditions, and the altered salivary contents may constitute a risk factor for major oral health issues [11,12].

There are two types of acinar cells—serous and mucous acinar—in the salivary glands. Serous acini are composed of typically 8–12 pyramid-shaped cells containing many secretory granules in the apical cytoplasm, a well-developed rough-endoplasmic reticulum (rough-ER), and pronounced round nuclei in the middle of the cytoplasm. They secrete serous saliva, which contains digestive α-amylase (AMY1A), a protein that is crucial for food digestion [13,14]. By contrast, mucous acinar cells are larger than serous acinar cells and have flat nuclei towards the basal cell surface due to large numbers of mucin granules aggregated in the apical cytoplasm [13,14]. Mucous saliva contains mucins that are important for lubrication and oral health [13,14].

The salivary glands secrete saliva rich in salivary proteins, the diverse functions of which maintain oral health by providing lubrication, initiating digestion, and offering first-line immunity. Therefore, disruption of salivary gland functions quickly results in widespread deterioration of oral health. Despite the important physiological functions of salivary proteins, we know very little about the regulatory mechanism(s) of exocytosis in the salivary glands. A detailed understanding of the mechanism(s) by which exocytosis is regulated will provide new knowledge of its key function(s), not only in the salivary glands but also in other secretory organs, including the lacrimal and mammary glands, the pancreas, and prostate, in physiological and pathological conditions. Ultimately, this approach will identify novel targets for therapeutics and contribute to new diagnostic tools for identifying exocytosis defects in at-risk populations such as those with high cholesterol levels or who have high-cholesterol diets.

Sjögren’s syndrome is an autoimmune disorder characterized by lymphocytic infiltration of the exocrine glands, mainly the salivary and lacrimal glands, which results in reduced secretory functions and oral and ocular dryness [15,16]. The diagnosis is based on a combination of symptoms, physical examination, and blood tests (American-European Consensus Group, AECG, 2002; Sjögren’s International Collaborative Clinical Alliance, SICCA, 2012). While the pathogenesis of Sjögren’s syndrome remains elusive, various factors, including environmental, genetic and hormonal factors, seem to be involved, and either immune cells or exocrine gland cells are primarily damaged or disorganized to induce inflammation in the salivary and lacrimal glands [17,18,19].

## 2. The Exocytosis Process

Exocytosis is a dynamic secretion process consisting of vesicle trafficking, tethering, docking, priming, and fusion [6,30]. After proteins are folded in the ER, they leave this organelle inside vesicles coated with the coat protein complex II (COPII) towards the Golgi apparatus. Through the *trans*-Golgi network, proteins are transported to various destinations by a variety of mechanisms. For example, the destination of the different proteins is determined by the molecule coats on a vesicle, by actin-dependent motors (Myosins), and by microtubule-dependent motors (Kinesins and Dyneins) [31,32]. During intracellular transportation, RAB GTPases and their effectors regulate the secretory vesicle movement throughout the cytoskeleton [33].

**Figure 1:**
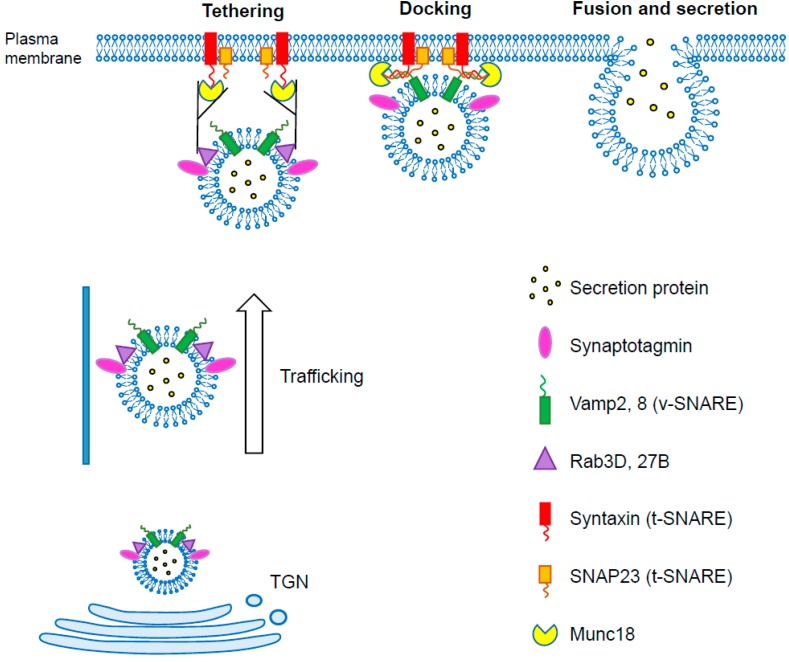
The exocytosis process. Exocytosis consists in secretory vesicle trafficking, docking, priming, and membrane fusion. These processes are regulated by soluble *N*-ethylmaleimide-sensitive factor attachment protein receptor (SNARE) proteins and various accessory proteins. TGN, *trans*-Golgi network.

Soluble *N*-ethylmaleimide-sensitive factor attachment protein (SNAP) receptor (SNARE) proteins, such as vesicle-associated membrane proteins (VAMPs), are present in the secretory vesicle membrane (Figure 1). The secretory vesicles are transported from the *trans*-Golgi network via actin- and microtubule-based molecular motor proteins towards the plasma membrane. Once the secretory vesicles arrive to the plasma membrane, tethering factors (e.g., exocyst) promote *trans*-SNARE formation through interactions between RABs, MUNC18, and SNARE proteins [34]. Docking is the process during which the vesicle and plasma membrane line up in a fusion-ready state. Following docking, the plasma membranes fuse with the secretory vesicles to create a small pore that grows larger until exocytosis occurs. Membrane fusion and pore opening processes are similar to zippering [35,36,37].

### SNARE Proteins

SNARE proteins have been identified as regulatory molecules that are crucial for exocytosis [38]. SNARE proteins are small, abundant tail-anchored proteins that are often post-translationally inserted into the membranes via a C-terminal transmembrane domain [39]. They have a similar structure in a cytosolic domain that is known as the SNARE motif, which is characterized by coiled-coil sequences of 60–70 residues and heptad repeats [40]. SNARE proteins are subcategorized into *v*-SNAREs (proteins present in vesicles) and *t*-SNAREs (target proteins syntax) [40,41,42]. The *v*-SNAREs and *t*-SNAREs assemble in a twisted parallel four-helical bundle/four-strand coiled-coil structure (trans-SNARE complex) to form a complex that is incorporated into the membranes of transport vesicles during exocytosis [40]. The four-helix bundle is extremely stable and enables the vesicles to fuse with the plasma membrane, thus allowing the release of the vesicle contents [35,40]. SNAREs are also differently classified into R-SNAREs and Q-SNAREs. R-SNAREs act as *v*-SNAREs and contribute with an arginine (R) residue in the formation of the zero ionic layer which is the main site of interaction in the assembled core SNARE complex, whereas Q-SNAREs act as *t*-SNAREs and contribute with a glutamine (Q) residue in the formation of the zero ionic layer in the assembled core SNARE complex [38,43].

## 3. *v*-SNAREs

### 3.1. Vamp8/Endobrevin

Vesicle-associated membrane protein 8 (VAMP8; aka Endobrevin) was first described as an R-SNARE of the early endosomal compartments [44], but recent studies indicate that it is an important component in exocytosis [45]. VAMP8 is enriched in zymogen granular membranes of pancreatic acinar cells and is crucial for regulated secretion [46]. Although mice deficient for *Vamp8* (*Vamp8^−/−^*) are normal at birth, they display growth retardation and severe defects in the pancreas [46]. In the salivary glands as well as the lacrimal glands and other exocrine organs, *Vamp8^−/−^* acinar cells show accumulation of secretory granules and increased levels of intracellular amylase and carbonic anhydrase VI, due to failure to interact with *t*-SNARE, Syntaxin-4, and SNAP-23 [20,46]. VAMP8 is also expressed in the apical plasma membrane in acinar cells and ductal epithelial cells in the major salivary glands and lacrimal glands [20]. In addition, *Vamp8^−/−^* mice show reduced mucin secretion in airway goblet cells after stimulation with interleukin-13 (IL-13) [47]. VAMP8 expression in the lacrimal glands is altered, from the apical to the basal side, in individuals with dry eyes [48]; in Sjögren’s syndrome, the expression of the gene and protein is decreased in the acinar cells of labial salivary glands. While VAMP8 is expressed in the apical cytoplasm in healthy individuals, it is expressed in the entire cytoplasmic in patients with Sjögren’s syndrome [49]. Thus, VAMP8 plays a crucial role in exocytosis in various exocrine glands.

### 3.2. Vamp2/Synaptobrevin2

Vesicle-associated membrane protein 2 (VAMP2; aka Synaptobrevin) is a 19-kDa small protein that is crucial for vesicle fusion [41]. VAMP2 consists of a short NH_2_-terminal sequence (called SNARE motif) and a COOH-terminal transmembrane region [50]. Mice deficient for *Vamp2* exhibit a rounded body shape and a shoulder hump that is caused by excessive brown fat in the upper back [51]. While *Vamp2^−/−^* mice develop normally, they exhibit defects in Ca^2+^-triggered exocytosis of synaptic vesicles in neurons and chromaffin cells [51,52]. VAMP2 localizes at the secretory granular membrane in rat parotid gland acinar cells and regulates cAMP-dependent amylase secretion through interactions with Syntaxin-4 in the plasma membrane [53,54,55]; by contrast, VAMP2 interacts with Complexin-2 at the apical secretory granular membrane in rat pancreatic acinar cells [56,57]. In human submandibular glands, VAMP2 localizes at the apical plasma membrane of acinar cells [58].

### 3.3. Synaptotagmins

Synaptotagmins (SYT) are a family of vesicle membrane proteins distributed in both neuronal and non-neuronal tissues that comprise at least 15 isoforms in mammals [59,60,61]. SYT members have two C2 domains (C2A and C2B domains) that are important for Ca^2+^-dependent binding with the plasma membrane and SNARE proteins. Based on Ca^2+^ dependency in binding with phospholipids, SYT proteins are classified as Ca^2+^-dependent (SYT-1, -2, -3, -5, -6, -7, -9, and -10) and Ca^2+^-independent types. *Syt1 and Syt3* are expressed in pancreatic acinar cells in mice and rats [57,62], whereas *Syt1*, *-3*, *-4*, *-7*, and *-11* are expressed in the acinar cells of rat parotid glands [63,64] and *Syt1*, *-2*, *-3*, *-4*, *-6*, and *-7* are expressed in the acinar cells of mouse parotid glands [63]. In the brain, *Syt1^−/−^* mice show a neurotransmitter secretion defect and synaptic transmission abnormality [65], and adrenal chromaffin cells exhibit reduced exocytosis in these mice [66]. *Syt2^−/−^* mice exhibit decreased and delayed neurotransmitter release at the synapse [67], and *Syt7^−/−^* mice show osteopenia because Cathepsin K secretion from osteoclasts and type I collagen secretion from osteoblasts are suppressed, resulting in decreased bone resorption and bone formation [68]. In addition, *Syt7^−/−^* mice show fibrosis in the skin and muscles and overactivation of the immune response, which is likely to cause autoimmune disorders [69]. Mice with a deficiency for either *Syt3*, *Syt4*, *Syt5*, *Syt6*, *Syt10*, or *Syt11* show normal phenotypes and behavior. These studies suggest that there is functional redundancy among several SYTs, which can compensate for the functions of absent proteins in several knockout mouse models.

## 4. *t*-SNARE

### 4.1. Syntaxins

Membrane fusion, opposed by repulsive forces between phospholipid bilayers, causes electrostatic repulsion of equally charged membrane surfaces. To overcome these energy barriers, specialized fusion proteins are required [37,70]. Syntaxins (STXs) are categorized into Q-SNARE proteins, which are characterized by a SNARE motif (coiled-coil α-helical structure), transmembrane domain (anchored C-terminal tail), and linker region [71]. The SNARE motif interacts with another molecule from the STX or SNAP family in order to form a *t*-SNARE complex. STX1A is expressed in presynaptic vesicles and forms a complex with SNAP25 and VAMP2 to induce synaptic exocytosis. Some STXs are expressed in exocrine acinar cells; for example, STX2 and STX3 localize at the apical plasma membrane, and STX4 is abundantly expressed in the plasma membrane in rat parotid glands. STX3 and STX4 form a complex with SNAP23 and VAMP8 in the apical membrane [55,72]. In human submandibular glands, STX2 is expressed in both the apical plasma membrane and cytoplasmic vesicles, and STX4 is expressed in the apical and basolateral plasma membranes [58]. In pancreatic acinar cells, STX4 is expressed in the basolateral plasma membrane while STX2 is present in the apical plasma membrane and zymogen granules, and STX-3, -7 and -8 are expressed in zymogen granules [73,74,75]. In terms of exocytosis, a few studies used *Stx* knockout mice. The pancreatic acinar cells from *Stx2^-/-^* mice show increased exocytosis because of a failure in the inhibition of secretory vesicle fusion to the plasma membrane mediated by STX3 and STX4 [21]. The *Stx4^−/−^* genotype is lethal before embryonic day 7.5 [76]. Mice with a heterozygous deletion of *Stx4* (*Stx4^+/−^* mice) show reduced secretion of glucose-stimulated insulin from β-cells in the pancreatic islets [77]; on the other hand, *Stx4* transgenic mice show increased insulin secretion [77]. In patients with primary Sjögren’s syndrome, STX4 gene and protein expression is decreased in labial salivary glands, while STX3 gene and protein expression is increased, compared to healthy individuals. The intracellular expression patterns of STX3 and STX4 also dramatically change in patients with Sjögren’s syndrome. While STX3 is normally localized in the apical region of acinar cells in labial salivary glands, STX3 is expressed in the entire cytoplasm and the basolateral plasma membrane in patients. STX4 localizes more abundantly at the basolateral plasma membrane than the apical plasma membrane in healthy individuals. In patients with Sjögren’s syndrome, while STX4 localizes at the basal plasma membrane, similar to healthy individuals, its expression is decreased in the apical and lateral plasma membranes [49]. Interestingly, the number of STX4-VAMP8 and STX3-SNAP23 complexes is increased in acinar cells in the labial salivary glands of the patients. The STX4-VAMP8 complex is present in the basolateral membrane in patients while no STX4-VAMP8 complex has been detected anywhere in healthy individuals. Moreover, co-localization of STX4 and RAB3D, a small GTPase, in mature secretory vesicles in the subapical region is altered from the apical to the basolateral plasma membrane in Sjögren’s syndrome patients. Corresponding to the disruption of expression and co-localization of SNARE complexes, secretory mucins (MUC5 and MUC7) are distributed not only in the cytoplasm but also in extracellular matrix in Sjögren’s syndrome [49,78]. This suggests that inadequate expression of SNARE complexes leads to aberrant mucin exocytosis in the basal region, resulting in the accumulation of mucin in the extracellular matrix.

### 4.2. SNAPs

The SNAP25 family of proteins (SNAP25, SNAP23, SNAP29, and SNAP47) is included in the group of Q-SNARE proteins and anchor to the cytosolic face of the plasma membrane [79]. SNAP25 is expressed in the plasma membrane of neuronal and endocrine cells and forms a SNARE complex with STX1 and VAMP2. SNAP23 is a non-neuronal homolog of SNAP25 that is expressed in many cell types. In human submandibular glands, SNAP23 is specifically expressed in the apical membrane and the basolateral plasma membrane [58], but in rat parotid glands it can be detected in the apical plasma membrane and the intracellular membrane and interacts with STX3, STX4, VAMP2, and VAMP3 [72]. In rat pancreatic acinar cells, SNAP23 is expressed in the entire plasma membrane and zygomatic vesicles and forms a complex with SYN2 and VAMP2 in order to regulate exocytosis in the apical plasma membrane [80,81]. While SNAP23 is present in the plasma membrane of acinar cells in labial salivary glands of healthy individuals, SNAP23 expression is absent in the apical plasma membrane and remarkably decreased in the lateral plasma membrane in patients with Sjögren’s syndrome, although its expression is not altered at the level of the basal plasma membrane [49]. *Snap23^−/−^* mice die at the pre-implantation embryonic stage [82], and hippocampal neurons from heterozygous *Snap23* knockout mice show impaired recycling of *N*-methyl-d-aspartate (NMDA) receptors and decreased expression of NMDA receptors on the surface of the cells [83]. *Snap25^−/−^* mice show decreased insulin secretion from β-cells and glucagon from α-cells of islets as well as decreased glutamine release from synapses [84]. The exocytosis phenotype in the salivary glands and lacrimal glands of these mice has not been reported.

### 4.3. RAB

RAB proteins are members of the Ras superfamily of small GTPases (21–25 kDa). Until now, at least 70 RAB proteins have been reported in mammals [85]. RAB GTPases are anchored at the cytosolic face of the intracellular membrane and play important roles in membrane trafficking such as vesicle formation, movement along the cytoskeleton, docking, priming, and membrane fusion processes during exocytosis. RAB proteins switch between active (GTP-bounding) and inactive (GDP-bounding) forms via GDP/GTP exchange factors (GEFs; activators) and GTPase-activating proteins (GAPs; inactivators). The GDP-bounding form is delivered to the appropriate membrane by RAB escort proteins and functions as a regulator of intracellular trafficking with RAB effector molecules, which are heterogeneous protein complexes involved in multiple functions, such as cargo selection, vesicle budding, movement throughout the cytoskeleton, tethering, and membrane fusion [86,87]. Previous studies indicate that several RAB proteins (RAB-1B, -3D, -4, -5A, -11, -26, -27A, and -33A), escort proteins, effectors (Slp4-a/granuphilin-a, Slac2-c/melanophilin, and Noc2/rabphilin 3A-like), and GEFs are expressed in rat parotid gland acinar cells [88,89,90,91,92,93,94,95,96,97,98]. *Rab3d*, *Rab11a*, *Rab27a*, and *Rab27b* are expressed in lacrimal gland acinar cells [24,99,100]. For example, RAB3A-3D is involved in regulated exocytosis, and RAB3D, but not RAB3A-C, is highly expressed in subapical secretory granules in acinar cells of the parotid glands, lacrimal glands, and the pancreas, and in synaptic vesicles in the brain [95,96,101]. While *Rab3d* null mice are not affected in terms of amylase secretion and number of secretory granules in acinar cells of the pancreas and parotid glands, the size of secretory vesicles is larger in *Rab3d* null mice compared to controls because of a failure in the maturation of secretory vesicles [22]. On the other hand, in the lacrimal glands of *Rab3d* null mice the total protein amount in tears is decreased [23]. Mice overexpressing *Rab3d* (*Rab3d* transgenic mice) show enhanced amylase secretion in pancreatic acinar cells under stimulation [102]. Mice with a deletion of *Rab27a* (*Ashen* mice) or *Rab27b* show slightly increased secretion of total protein in tears [23]. The number of secretory vesicles is significantly decreased in *Rab27b^−^*^/*−*^ and *Rab27a^−/−^*; *Rab27b^−/−^* double knockout mice compared to control mice. *Rab27a^−/−^* mice, as well as lacrimal gland acinar cells transfected with a dominant negative form of *Rab27b*, show a reduction in syncollin-GFP secretion in response to carbachol stimulation [24]. Interestingly, *NOD* mice, a mouse model for Sjögren’s syndrome, show decreased *Rab3d* expression and altered distribution of RAB3D from the subapical vesicles to the basolateral region [23,103]. Both *Rab3d^−/−^* and *NOD* mice show elevated expression and enzymatic activity of Cathepsin S, a secreted protease, in lacrimal gland acinar cells; by contrast, *Rab27a^−/−^* and *Rab27b^−/−^* mice show decreased Cathepsin S activity [23,104]. The total protein amount is inversely correlated with Cathepsin S activity in these mice [23]. Patients with Sjögren’s syndrome show significant reduction in RAB3D expression, but not RAB8A, as well as the mislocalization of RAB3D from the apical to the basolateral region in salivary gland acinar cells [49,105] and in lacrimal gland acinar cells [48]. The enzymatic activity of Cathepsin S is upregulated in tears from Sjögren’s syndrome patients, as seen in *Rab3d^−/−^* and *NOD* mice [106,107]. Taken together, RAB expression seems to be altered in the exocrine glands of patients with Sjögren’s syndrome.

## 5. Accessory Proteins

### 5.1. MUNC18

MUNC18 (aka STXBP) is a Sec1/Munc18-like (SM) protein that includes three lobes folding into a clasp structure. MUNC18 binds to Syntaxin, is required for intermembrane trafficking in exocytosis, and is composed of six members [MUNC18-1 (aka STXBP1) to MUNC18-6 (aka STXBP6)]. MUNC18-1 is specifically expressed in the brain, and *Munc18-1* null (*Munc18-1^−/−^*) mice die at birth as a result of cell autonomous neuronal degeneration [108]. Mice with a conditional deletion of *Munc18-1* in the cerebellum (*Munc18-1^fl/fl^; Pcp2-Cre* mice) show ataxia by 8 weeks old [109]. MUNC18-2 is expressed in acinar cells in the parotid glands and the pancreas and interacts with Syntaxin-2 in rats [110,111]. Mice deficient for *Munc18-2* show decreased mucin secretion in airway epithelial cells and decreased histamine secretion in mast cells [112,113]. MUNC18-3 interacts with Syntaxin-4 in the apical membrane of parotid gland acinar cells and the basolateral membrane of pancreatic acinar cells for the regulation of amylase secretion in rats [114,115]. *Munc18-3* heterozygous mutant mice show reduced insulin secretion in response to glucose stimulation in islets through granular delocalization to the plasma membrane [116].

### 5.2. NOC2

NOC2 (aka rabphilin 3A-like) is a putative RAB GTPase effector for RAB2A, RAB3, RAB8, and RAB27 that is widely expressed in endocrine and exocrine cells [25,117,118]. For example, *Noc2* is expressed in acinar cells of the pancreas and salivary glands, granular convoluted duct cells in the submandibular glands, gastric chief cells, Brunner glands in the duodenum, Paneth cells in the jejunum, and mucous cells in the stomach. These cells are enlarged in *Noc2^−/−^* mice due to the accumulation of enlarged secretory vesicles [26]. The increased size of secretory vesicles in *Noc2^−/−^* mice is similar to that in *Rab3d^−/−^* mice [25]. *Noc2^−/−^* mice release less insulin from the pancreatic islets and less amylase from pancreatic acinar cells compared to wild-type controls. Thus, NOC2 plays a crucial role in regulated exocytosis in both endocrine and exocrine cells.

### 5.3. SEC

Newly synthesized proteins are delivered to specific cellular organelles or the plasma membrane by cargo, which is sorted by COPII vesicles during transportation from the ER to the Golgi apparatus. The COPII coat contains a small GTPase SAR1 and two cytosolic protein complexes, Sec23/Sec24 and Sec13/Sec31. GDP-bound inactivated Sar1 interacts with Sec12, a membrane-bound GEF, and is exchanged with the GTP-bound active form. Activated Sar1 binds to a Sec23/Sec24 complex in the ER membrane, and finally the budding vesicle recruits a Sec13/Sec31 complex and then the COPII vesicle is released from the ER membrane [119]. SEC23 plays a role in COPII vesicle tethering to the Golgi [27,120], and *Sec13* suppression reduces the secretion and deposition of collagen fibrils and disturbs budding from the ER in Hela cells and human fibroblasts [121]. In addition, suppression of *Sec13* in zebrafish results in craniofacial anomalies such as absence of Meckel’s cartilage and disorganization of the neurocranium [121]. The phenotype is similar to that of *crusher* zebrafish, which has a mutation in *Sec23a*, and in patients with cranio-lenticulo-sutural dysplasia (CLSD) with mutations in *SEC23A* [122,123,124,125,126]. These studies suggest that SEC23A plays a role, not only in the capture of cargo, but also in the coupling of Sec23/24 and Sec13/31, which are both important for craniofacial development [121,126]. Patients with mutations in *SAR1B* have Chylomicron retention disease (CMRD), a hypocholesterolemic disorder characterized by lipid malabsorption. Mutations in *SAR1B* result in impaired chylomicron trafficking from the ER to the Golgi and cause accumulation of prechylomicron-containing vesicles in the cytoplasm of enterocytes [127,128]. Mutations in *SEC23B* have been reported in congenital dyserythropoietic anemia type II (CDAII) [129,130], and mice deficient for *Sec23b* (*Sec23b^gt/gt^* mice) display developmental defects in pancreatic acinar cells such as degeneration of acinar cells, absence of zymogene granules, accumulation of exocrine proteins in the ER, and dilated ER since the contents of the zymogen granules cannot exit from the ER via COPII vesicles [27]. *Sec23b^gt/gt^* mice also show reduced body and pancreas size compared to controls at birth while there is no defect in hematopoiesis/number of red blood cells and skeletal development, which can be seen in CDAII and CLSD, respectively. In addition to pancreatic acinar cells, other exocrine glands (e.g., submandibular, sublingual, and nasal glands, gastric mucus epithelium, and goblet cells) are similarly affected in *Sec23b^gt/gt^* mice, including degeneration of acinar cells, dilated ER, and reduced or no secretory vesicles in these glands [27]. Mice with a pancreatic acinar cell-specific deletion of *Sec23b* (*Sec23b^fl/fl^*; *Ela-CreErT*) show phenotypes similar to those of *Sec23b^gt/gt^* mice. The amylase amount in total lysate from pancreatic cells is slightly decreased in these *Sec23b* conditional knockout mice but is not affected in the plasma [28,29]. These results indicate that the inhibition of the early steps of trafficking results in the reduction in number of secretory vesicles.

## 6. Other Potential Regulators

### 6.1. Lipid Metabolism

It has long been appreciated that individuals with metabolic syndromes and unhealthy diets, including those with a high fat content, are at risk of a variety of oral diseases [131,132]. It has been suggested that salivary protein content is altered in patients with diabetes and obesity, who present a higher frequency of oral health issues [12,133,134,135,136,137,138,139,140]. A recent study indicates that altered salivary composition induced by high fat-diet obesity is restored with normal diets in Wistar rats [141]. Moreover, animal studies suggest that obesity induced by high-fat diets or atherogenic cholesterol-rich diets triggers periodontal disease as well as alveolar bone loss [142,143,144]. A low-fat, high-fiber diet, however, improves periodontal diseases [145].

### 6.2. Neuronal Regulation

The secretion of protein and water in the salivary glands is regulated by parasympathetic and sympathetic nerves. Denervation by parasympathectomy and sympathectomy results in reduction of saliva secretion, and subsequently atrophy, in animal models and humans [146,147,148,149,150]. However, it remains unknown whether denervation or neuronal dysfunction causes the Sjögren’s syndrome-like phenotype. Since synaptic vesicle exocytosis is crucial for neural function, the link of Sjögren’s syndrome and neuronal dysfunctions should be investigated in the future.

## 7. Conclusions

Salivary proteins (amylase, mucin, and immunoglobulin) are functionally important for aiding digestion, providing lubrication, preventing oral infections, and maintaining overall oral health. Salivary proteins are secreted via exocytosis by acinar cells in the salivary glands. Despite its importance, the regulatory mechanism of salivary exocytosis has been elusive because of: (1) the lack of tools for tracking exocytosis, and (2) the lack of animal models with exocytosis defects in the salivary glands. The current limitation of mouse models is that almost all models for Sjögren’s syndrome (or related dry mouth syndromes) have mutations and/or defects in the immune cells themselves [19]. Defects in each step of vesicle trafficking and membrane fusion processes may result in a failure of salivary protein secretion to the oral cavity as well as in the accumulation of secretory granules in acinar cells that may lead to the death of acinar cells. This might, in fact, be a trigger of inflammatory responses in pathogenesis and progression of Sjögren’s syndrome. Further studies will contribute to the development of new tools to track exocytosis and to the development of new mouse models to understand the mechanism(s) of exocytosis in healthy individuals and in patients with Sjögren’s syndrome.

## Figures and Tables

**Table 1:** Phenotype in mice with deficiencies in genes related to the exocytosis process.

Gene Name	Mutant Mouse Phenotype			Reference
	Salivary Acinar Cells	Lacrimal Acinar Cells	Pancreatic Acinar Cells	
*Vamp8*	Accumulation of secretory vesicles	Accumulation of secretory vesicles	Accumulation of secretory vesicles	[20]
*Syntaxin2*	Not studied	Not studied	Increased exocytosis	[21]
*Rab3d*	Enlarged secretory vesicles	Enlarged secretory vesicles	Enlarged secretory vesicles	[22,23]
		Decreased total protein amount in tears		
*Rab27a*	Not studied	Increased total protein amount in tears	Not studied	[23,24]
*Rab27b*	Not studied	Reduced number of secretory vesicles	Not studied	[23,24]
		Increased total protein amount in tears		
*Noc2*	Accumulation of enlarged secretory vesicles	Not studied	Accumulation of enlarged secretory vesicles	[25,26]
*Sec23b*	Gland degeneration	Not studied	Gland degeneration	[27,28,29]
	Absent or reduced number of secretory vesicles		Absent or reduced number of secretory vesicles	
